# Self-Healing Polymers

**DOI:** 10.3390/polym14112261

**Published:** 2022-05-31

**Authors:** Alexander S. Novikov

**Affiliations:** 1 Saint Petersburg State University, Universitetskaya Nab., 7/9, 199034 Saint Petersburg, Russia; a.s.novikov@spbu.ru; 2 Peoples’ Friendship University of Russia (RUDN University), Miklukho-Maklaya St., 6, 117198 Moscow, Russia

Self-healing polymers are synthetic or artificially-created substances that have the built-in ability to automatically repair damages to themselves without any external diagnosis of the problem or human intervention. Generally, materials will degrade over time due to damage incurred during operation, environmental conditions or fatigue. Cracks and other types of damage on macro- and microscopic levels have been shown to change acoustical, electrical and thermal properties of polymers, and the propagation of cracks can lead to the eventual failure of the material. In general, cracks are hard to detect at an early stage, and manual intervention is required for periodic repairs and inspections. In contrast, self-healing polymers counter degradation through the initiation of a repair mechanism that responds to the microdamage. Some self-healing polymers are classed as smart structures and can adapt to various environmental conditions according to their sensing and actuation properties. Although the most common types of self-healing materials are elastomers or polymers, self-healing covers all classes of materials, including cementitious substances, ceramics and metals. Healing mechanisms vary from an intrinsic repair of the polymer to the addition of a repair agent contained in a microscopic vessel. For a polymer to be strictly defined as autonomously self-healing, it is necessary that the healing process occurs without human intervention. Self-healing polymers may, however, activate in response to an external stimulus (e.g., pressure, temperature, light) to initiate the healing processes. A polymer that can intrinsically correct damage caused by normal usage could prevent costs incurred by material failure and lower the costs of a number of different industrial processes through the longer lifetime of parts and the reduction of inefficiency caused by degradation over time. The field of self-healing polymers is related to biomimetic materials as well as to other novel materials and surfaces with the embedded capacity for self-organization, such as self-cleaning and self-lubricating materials. 

The following modern directions in the development of self-healing polymers are noted: cross-linked polymers, polymerization of multifunctional monomers, thiol-based polymers, poly(urea-urethane), vitrimers, microcapsule healing, 1D, 2D and 3D vascular-based polymeric systems, hollow tube polymers, discrete channels and interconnected networks in polymers, carbon nanotube networks, sacrificial thread stitching, self-healing coatings, self-healing cementitious materials, self-healing ceramics, self-healing metals, self-healing organic dyes, the self-healing of ice and bio-based healing.

The aim of this Special Issue is to highlight and overview modern trends and attract the attention of the scientific community to the problem of self-healing polymers. All types of papers (reviews, mini-reviews, full papers, short communications, technical notes, highlights, etc.) are welcome for consideration.

